# Editorial: Nursing has always been a form of resistance

**DOI:** 10.1177/17449871251347858

**Published:** 2025-07-02

**Authors:** Pernilla Garmy

**Affiliations:** Faculty of Health Sciences, Kristianstad University, Sweden

*‘There are things you have to do, even if they are dangerous’, Jonathan said.*

*‘Why?’ I asked.*

*‘Because if you don’t, you’re not really human’.*

–Astrid Lindgren, *The Brothers Lionheart*

These words, spoken by a boy to his younger brother in Astrid Lindgren’s *The Brothers Lionheart*, have stayed with generations of readers. The original Swedish phrase – *‘Annars är man ingen människa utan bara en liten lort’* – uses the word *lort*, a childlike term for dirt or filth. But in context, it speaks to something deeper: the courage it takes to act with humanity, even when it’s hard. To be passive in the face of fear is not simply cowardice — it’s a type of moral failure.

This idea – that we must act even when afraid – resonates deeply today. In her recent editorial *‘We Were Made for These Times’*, Sally Thorne (2025) reminds us that nurses are indeed made for these times. Not because we are fearless, but because we understand the ethical imperative to act – again and again – even in the face of political regression, social fragmentation, and public mistrust in science. In moments when despair feels overwhelming and the future uncertain, her words offer more than analysis; they offer resolve, courage, and a reaffirmation of purpose.

Thorne also draws on Clarissa Pinkola Estés’ *Letter to a Young Activist During Troubled Times* (2001/2016), where Estés writes that profound change does not require everyone on Earth – it takes only a small, determined group who refuse to give up. Transformation, she suggests, comes from ‘an accumulation of acts – adding, adding more, continuously’. In that spirit, nursing must be seen not only as care, but as quiet, persistent resistance – rooted in justice, equity, and the enduring good.

## Resistance in everyday practice

To me, resistance as a nurse means caring for fellow human beings regardless of their political beliefs or background. It also means being vigilant about my own biases, ensuring they do not interfere with my capacity to treat others with respect and compassion.

As a researcher, resistance means addressing complex, sometimes uncomfortable topics – and making sure those who are often excluded from research are brought into its centre. This includes individuals marginalised due to sexual orientation, ethnicity, age, disability, or other forms of social vulnerability.

Resistance often happens in subtle moments: staying present in a difficult conversation, listening to a young person express existential loneliness, or exploring children’s fears around sleep and anxiety. It means continuing to study inequality, to amplify the voices of LGBTQ+ individuals, and – in my role as an editor – to publish papers that challenge the status quo, even when they swim against today’s rising tides of intolerance.

## The power of person-centredness

Diversity is essential in both research and practice – not only because it is a matter of justice, but because it enriches and deepens our work. It is through learning from others that we grow.

Person-centred nursing is grounded in ethical and humanistic values (McCance and McCormack, 2025). It is about recognising both ourselves and others as human beings of inherent worth and dignity. It is about seeing individuals not as problems to be solved, but as people to be understood and supported. In this sense, person-centred care becomes a quiet form of resistance – a refusal to reduce anyone to their diagnosis, circumstance, or demographic.

This orientation also calls on us to work across boundaries – especially the one that too often separates research from practice. Practitioners highlight the most urgent questions and show what knowledge needs to do to be useful. Researchers, in turn, must take responsibility for ensuring that findings are accessible, relevant, and actionable.

## Nursing as ethical action

Nursing has always been grounded in human need – not in political affiliation. Our epistemological foundation points clearly to an ethical commitment: the belief in the equal value and dignity of all people.

Nursing is both science and art. It is grounded not only in evidence, but also in the ability to be present, to notice, and to act. When we stand up for what research tells us works, when we approach our work with critical awareness, and when we defend the rights and dignity of every person – we are not only caring, we are engaging in ethical action. We are contributing to what Estés calls the *enduring good*.

## Let us continue

What we do – as nurses, as researchers, as colleagues – matters. In a world often difficult to navigate, it is worth returning to what Aaron Antonovsky (1987) called a *sense of coherence* – composed of comprehensibility, manageability, and meaningfulness. These three pillars help us understand the world, believe we can manage its challenges, and find purpose in what we do.

Meaning does not always announce itself in dramatic moments. Often, it is found in the small, the quiet, the steady. It lives in a well-timed question, a research finding shared with care, a conversation that changes someone’s outlook, or a patient who feels truly seen.

Let us continue. With presence. With integrity. With courage. We want to be fully human — not by avoiding fear, but by meeting it with care and conviction.

Nursing has always been a form of resistance.
